# Utilization of social media in floods assessment using data mining techniques

**DOI:** 10.1371/journal.pone.0267079

**Published:** 2022-04-25

**Authors:** Qasim Khan, Edda Kalbus, Nazar Zaki, Mohamed Mostafa Mohamed

**Affiliations:** 1 Civil and Environmental Engineering Department, United Arab Emirates University, Al Ain, United Arab Emirates; 2 National Water Center, United Arab Emirates University, Al Ain, United Arab Emirates; 3 Department of Computer Science and Software Engineering, United Arab Emirates University, Al Ain, United Arab Emirates; Bristol University/Remote Sensing Solutions Inc., UNITED STATES

## Abstract

Floods are among the devastating types of disasters in terms of human life, social and financial losses. Authoritative data from flood gauges are scarce in arid regions because of the specific type of dry climate that dysfunctions these measuring devices. Hence, social media data could be a useful tool in this case, where a wealth of information is available online. This study investigates the reliability of flood related data quality collected from social media, particularly for an arid region where the usage of flow gauges is limited. The data (text, images and videos) of social media, related to a flood event, was analyzed using the Machine Learning approach. For this reason, digital data (758 images and 1413 video frames) was converted into numeric values through ResNet50 model using the VGG-16 architecture. Numeric data of images, videos and text was further classified using different Machine Learning algorithms. Receiver operating characteristics (ROC) curve and area under curve (AUC) methods were used to evaluate and compare the performance of the developed machine learning algorithms. This novel approach of studying the quality of social media data could be a reliable alternative in the absence of real-time flow gauges data. A flash flood that occurred in the United Arab Emirates (UAE) from March 7–11, 2016 was selected as the focus of this study. Random forest showed the highest accuracy of 80.18% among the five other classifiers for images and videos. Precipitation/rainfall data were used to validate social media data, which showed a significant relationship between rainfall and the number of posts. The validity of the machine learning models was assessed using the area under the curve, precision-recall curve, root mean square error, and kappa statistics to confirm the validity and accuracy of the model. The data quality of YouTube videos was found to have the highest accuracy followed by Facebook, Flickr, Twitter, and Instagram. These results showed that social media data could be used when gauge data is unavailable.

## Introduction

The arid climate of the Arabian Peninsula presents unique challenges for flood management [1]. Floods occurring after high-intensity rainfall events are a significant concern as flooding has considerable impacts including damage to infrastructure and loss of life [2]. Attempts to mitigate the impact of such incidents are, therefore, crucial especially with the predictions of increased climatic extremes associated with global climate change.

Rapid urbanization has altered the hydrological characteristics of land use, especially in the United Arab Emirates (UAE) [3]. The accumulation of surface water as a result of short-term rainfall is observed in the eastern and northern parts of the UAE [3, 4]. Flash floods in the UAE generally occur between November and March, where the mean runoff is about 120 Mm^**3**^ per year [5]. In the UAE, flow monitoring started in 1975 with seven flow gauges, which was increased to 21 in 1998 but then reduced to 10 flow gauges in 2005, and remained unchanged since then. For comparison, the City of Auckland in New Zealand operates 49 flow gauges over an area roughly the same size of Dubai [6].

To overcome the problem of flow data scarcity, the volunteered geographic information (VGI) approach provides the opportunity to collect additional information at low cost. The idea is to involve the public in the collection of relevant data according to pre-set standards and guidelines. This is also known as user-generated content (UGI). The use of VGI in flood-related studies started recently [7], with researchers estimating flood damage from VGI [8] or mapping flood extents [9, 10].

Recent approaches take advantage of the wealth of data distributed through social networks. Social media messages that have a geographic reference can be considered VGI, as they can be used to analyze what is happening at a specific location [11]. Participation by citizens is implicitly volunteered as they do not distribute information for flood monitoring [12]. Various social media platforms have been used to extract and analyze flood-related information including Twitter [13–15], Facebook [15], Flickr [16], and YouTube [17]. Social media such as Twitter has also been used to study food-security related data during natural hazards [18].

The main advantages of these new data sources are (i) they can provide much denser geographical coverage compared to traditional sensor networks, and (ii) they directly record the impact of a flood on the human environment, as the users usually document personal observations and experiences [16]. For these reasons, majority of studies on the use of social media for flood monitoring have been conducted in the context of disaster response and flood damage assessment [13–15, 19–22].

Due to the absence of flow gauges and appropriate quality data in arid regions, the identification of other sources of data is essential. This study evaluates the use of social media data related to floods, which has never been studied before in the GCC region. The quality of social media data, in addition to the data of each individual social media platform, Twitter, Facebook, Instagram, YouTube, and Flickr, was investigated.

## Literature review

Using social media-derived flood information has great potential for hydrological research to improve flood management. Photos with geographical reference and time stamps taken at different times during a flood event can be used to estimate the development of the flood hydrograph and the flow rate (in conjunction with other technical data from the catchment). Videos can show the onset of a flash flood and the movement of the flood wave through the catchment. Such data can be valuable for calibrating flow models. However, only a few studies have examined the use of this type of data for hydrological research and modelling [23–25]. Even fewer studies have investigated the use of social media data for the development of monitoring records for ungauged water bodies [17]. Examples of such studies are presented below:

Barker and Macleod [26] studied Twitter data to monitor flood events using real-time river levels across the United Kingdom. A paragraph vectors and a logistic regression-based classifier was used in the development of a Twitter data mining pipeline. The flood data obtained were then integrated with a real-time environmental data to give stakeholders better understanding of the local conditions. Another study by Bischke et al. [27] used satellite images for flood detection and enriched the information by using images from Twitter to understand the severity of floods. Twitter images were pre-processed by removing duplicate images and then an algorithm was designed that can identify those images and the water level as a result of flood, secondly high-resolution satellite images were also studied for high flood level by automatically detection of water levels and generating flood map. To identify the relevancy of georeferenced social media messages from Twitter during any flood event, de Albuquerque et al. [11] used statistical analysis, for identification of spatial patterns in the flood-related tweets and combining it with authoritative data by analyzing a case study of the River Elbe Flood in Germany in June 2013. The results showed that Tweets within a spatial distance of 10 km has higher probability of being related to a specific flood incident. The approach of the “wisdom of the crowd” with the number of Tweets—reliable patterns in the data, for a certain event versus the monitored watershed activity was studied for Jakarta, Indonesia [19]. This approach is more applicable to areas where more social media users are active. The observed information about the georeferenced activity of Twitter users in an area was mapped on a Digital Elevation Model (DEM) with flood depth observations and then used to create flood maps. The tweets with water depth showed very good and reliable indication of flood in that area. Similar study, by mapping flood depth from social media data, was also conducted by Karmegam et al. [28], and the results were validated with real time flow data.

A more detailed study in Argentina, France, and New Zealand was conducted by Le Coz et al. [23], where a dedicated website of the Flood Chasers Project was developed. People were encouraged to upload images and videos of any flood events. These images were processed using PIV/PTV analysis tools for flood mapping, and flood estimation using videos of river flow velocity and discharge were modeled using Large Scale Particle Image Velocimetry (LSPIV), which is an effective technique for post-flood discharge estimation. The implementation of these tools provides potential of citizen science for flood risk assessment. Other such studies in literature has been reported that uses VGI focused on flood damage using dedicated platforms, such as PetaJakarta in Jakarta, QLF flood crisis map in Australia [29], and flooding points in Brazil [30].

Restrepo-Estrada et al. [31] studied the social media for rainfall-runoff estimations and forecasting floods using data from Twitter and authoritative data. They combined geospatial Twitter data with real-time authoritative flood values as an input for the Probability Distribution Model (PDM) and achieved 71% accuracy. Addition of social media data has increased the accuracy of PDM almost twice. Rosser et al. [22] studied the 2014 UK flood using Flickr posting activity, remote sensing, and topographic map data using a Bayesian statistical model to develop a probability map that shows the likelihood of the presence of floodwater.

Panigrahi et al. [32] used deep neural networks for prediction of floods in terms of seven parameters including rainfall, area, pressure, velocity, gauge, average temperature and average windspeed. Two rivers Daya and Bhargavi in India were studied using deep learning models for predicting discharge volume. Results indicated that Local Linear Radial Basis Functional Neural Network (LLBRFNN) performed well in prediction of flood levels with lowest Mean Square error (MSE) and Mean Absolute Percentage Error (MAPE). Same rivers were used in another study by Panigrahi et al. [33] where Cascaded Functional Link Artificial Neural Network (C-FLANN) was developed; Harmony Search (HS) and Differential Evolution (DE) was used to update the parameters of the model. Same seven atmospheric parameters were used to predict water flow in the rivers where the results showed that c-FLANN trained using HS gives better predictions of water level.

The major task in using social media for flood monitoring is the classification of large amounts of data in various formats (image, video, and text) and the extraction of relevant parameters from the data. Typically, machine-learning methods are used to aid in this task. For example, Huang et al. [34] used unsupervised machine learning method (clustering) to cluster text related to emergency based on similarity and combine it with supervised logistical regression to cluster posts based on different events. The selected methods for this study are described below.

ResNet is a pre-trained model for image classification [35]. The ResNet architecture is often used for feature extraction, image classification, image segmentation, and object detection. This method uses deep convolutional neural networks (CNNs) for identification and classification purposes.

Random forest is a machine-learning algorithm that is used for classification and regression. It is a method that constructs multiple decision trees. During the training time, individual decision trees are generated by randomly selecting the attributes in each node that determines the split. Each tree then gives its separate weights individually during classification. Random forests can handle data with outliers, and it can also handle missing values [36].

Naïve Bayes is a statistical classifier that provides conditional independence between predictors [36]. It is mainly based on the assumption that all predictors or attributes are conditionally independent, which is the reason for its naivety [37].

Sequential minimal optimization (SMO) is an implementation of a support vector machine (SVM) classifier in the Weka (Waikato Environment for Knowledge Analysis) platform. It is developed for numeric prediction and classifying data by the construction of an N-dimensional hyperplane that can separate data optimally into two categories [38]. SVM works well in text classification tasks as it has the ability to remove the need for feature selection [39].

Classification algorithm C4.5 produces a decision tree based on information theory. It uses the information entropy concept and uses a greedy technique to induce decision trees for classification [40]. It accepts nominal classes and is used in the construction of a decision tree from labelled training data that uses information entropy [37].

Earlier studies have focused on the development of a framework for social media activity in flooded areas, and some of them used dedicated volunteer geographic information to study such events on the hydrological catchment scale. However, there is a need to address the data quality of collected information from social media and the validity of such data for flood predictions, particularly for arid regions with patchy but intense rainfall and high risk of flash floods. This study, for the first time, analyses data obtained in an arid region from various social media platforms such as Twitter, Facebook, Instagram, Flickr, and YouTube. It validated social media data for flood monitoring. For this purpose, social media data from a period of heavy rainfall and the resulting flash flood events (March 7 to 11, 2016) were analyzed for the UAE, which is classified as an arid region. The validation of social media data for flood prediction and evaluation was performed based on the outputs of different machine learning classifiers. For this purpose, the area under the curve (AUC), root-mean-square error (RMSE), and kappa statistics were used. Data quality from Twitter, Facebook, Instagram, YouTube, and Flickr were also analyzed, and their model performance was assessed using AUC, RMSE, and kappa statistics.

## Data collection

**Fig 1:**
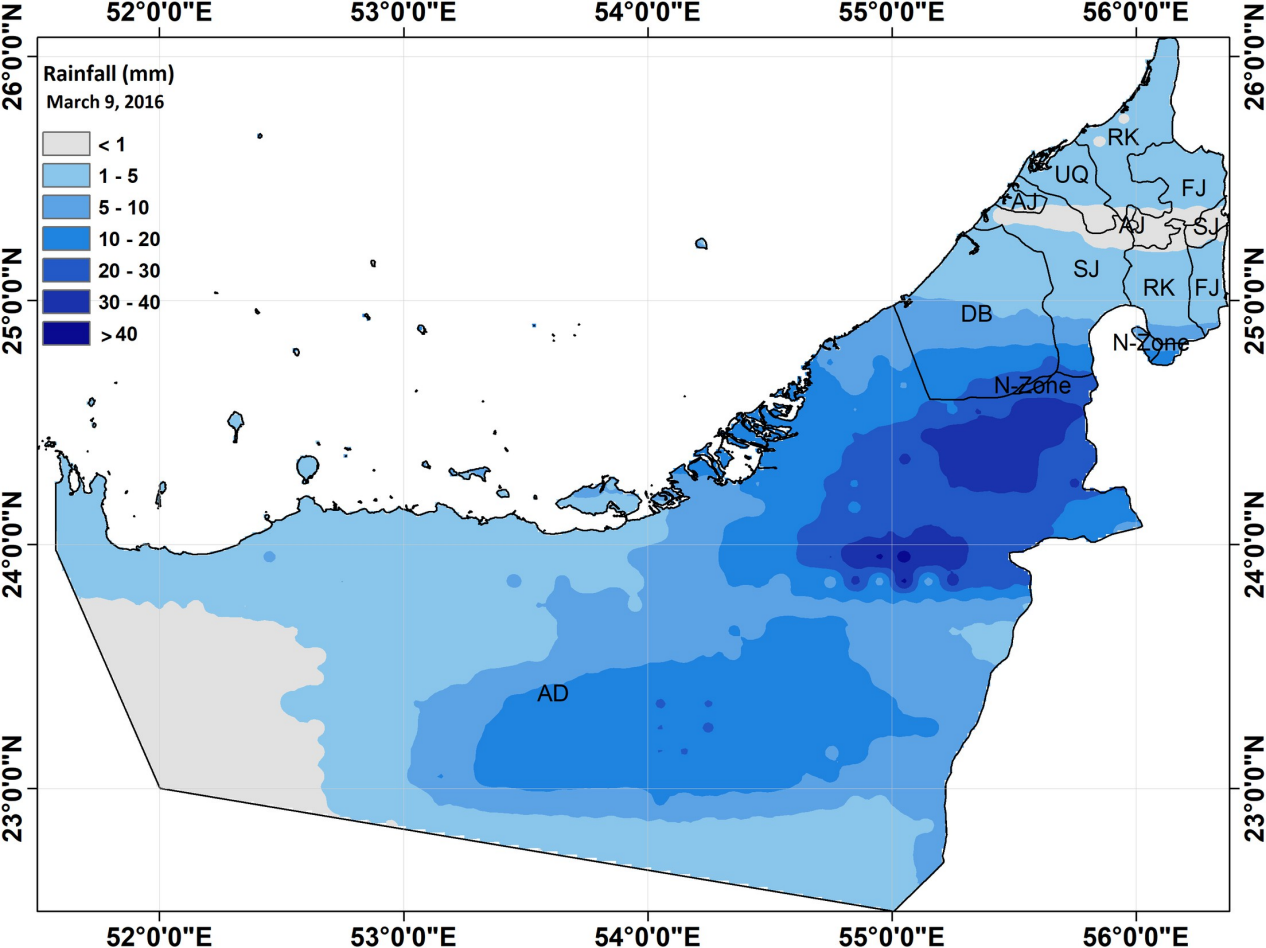
Study area United Arab Emirates showing highest rainfall event (data from global circulation model).

The UAE is located in a tropical dry region, where the Tropic of Cancer runs to the south of the country. The climate in the UAE is characterized by high temperatures throughout the year, [41]. Precipitation is scarce, and rainfall occurs only from December to April [42]. The average annual rainfall in 2015 was 87.4 mm, which decreased to 60.7 mm in 2016 (Fig 1).

Mild rainfall (1.2 mm) in 2016 occurred on March 8, which was followed by heavy rainfall (7.3 mm) on March 9 that brought the life to standstill in the UAE. To study the flash flood that occurred due to heavy rainfall in the UAE, 794 online posts were collected from different platforms using keywords and geographical queries given in Table 1. These hashtags were used in combination with the operators (‘AND’ and ‘OR’). To obtain relevant data on the UAE and seven different emirates, each geographical query (UAE, Abu Dhabi, Dubai, Al Ain, Sharjah, and RAK) was used with ‘AND’ followed by rain, flood, storm, and weather with ‘OR’ operator. Three different types of data were collected: text, images, and videos. To validate the data collected from social media, precipitation/rainfall data (mm/day) was obtained from the Global Precipitation Measurement (GPM), which is a climatic satellite that has the ability to detect and measure precipitation using advanced instruments. GPM data for the time frequency of day were obtained from the National Aeronautics and Space Administration (NASA) website (https://pmm.nasa.gov/data-access/downloads/gpm) for the period of March 7 to 11, 2016. A study by Mahmoud et al. [43] validated this data for the UAE for the period from 2015 to 2017 using ground values from the National Centre of Meteorology (NCM).

**Table 1:** Collected data from different social media platforms.

Platform	No. of posts	No. of images	No. of videos	#tags/Keywords (Search Date: 7^th^ to 11^th^ March 2016)
**Twitter**	261	88	16	#UAE AND #Rain OR #Flood OR #Storm OR #Weather
				#AbuDhabi AND #Rain OR #Flood OR #Storm OR #Weather
				#Dubai AND #Rain OR #Flood OR #Storm OR #Weather
**Facebook**	318	560	112	#AlAin AND #Rain OR #Flood OR #Storm OR #Weather
				#Sharjah AND #Rain OR #Flood OR #Storm OR #Weather
				#RAK AND #Rain OR #Flood OR #Storm OR #Weather
**Instagram**	112	66	44	#RainUAE OR #StormUAE #UAEWeather
				#RainAbuDhabi OR #AbuDhabiFlood OR #AbuDhabiWeather
				#RainDubai OR #DubaiFlood OR #DubaiWeather
				#RainAlAin OR #AlAinFlood OR #AlAinWeather
				#RainSharjah OR #SharjahFlood OR #SharjahWeather
				#RainRAK OR #RAKFlood OR #RAKWeather
				#RainSharjah OR #SharjahFlood OR #SharjahWeather
**Flickr**	25	21	0	UAE AND Rain OR Flood OR Storm OR Weather
				AbuDhabi AND Rain OR Flood OR Storm OR Weather
				Dubai AND Rain OR Flood OR Storm OR Weather
**YouTube**	78	0	78	AlAin AND Rain OR Flood OR Storm OR Weather
				Sharjah AND Rain OR Flood OR Storm OR Weather
				RAK AND Rain OR Flood OR Storm OR Weather
**TOTAL**	**794**	**735**	**250**	

The text, images and videos obtained from studied social media platform were then used to access the flash flood using machine-learning algorithms. The parameters used for carrying out simulations contain three major categories of text, images and videos. The text messages were converted to binary matrix which was then used as an input into the machine learning simulations. However, videos were first converted into frames, using python code (S1 Appendix), and those frames along with images were converted into features using VGG-16 architecture and then used as input data into machine learning model. Additionally, for simulation, 20-fold cross validation was used.

### Methodology

The pre-processing of data includes categorization of data into four classes: not relevant, rain, low flood, and high flood (Table 2). All the texts, images, and videos were categorized based on these classes. Duplicate images and videos were removed based on the user who first uploaded them on social media.

**Table 2:** Data classified into classes.

	Classes			
	Not Relevant	Rain	Low Flood	High Flood
**Images**	155	397	117	89
**Videos**	0	103	37	14
**Text**	122	448	91	44

**Fig 2:**
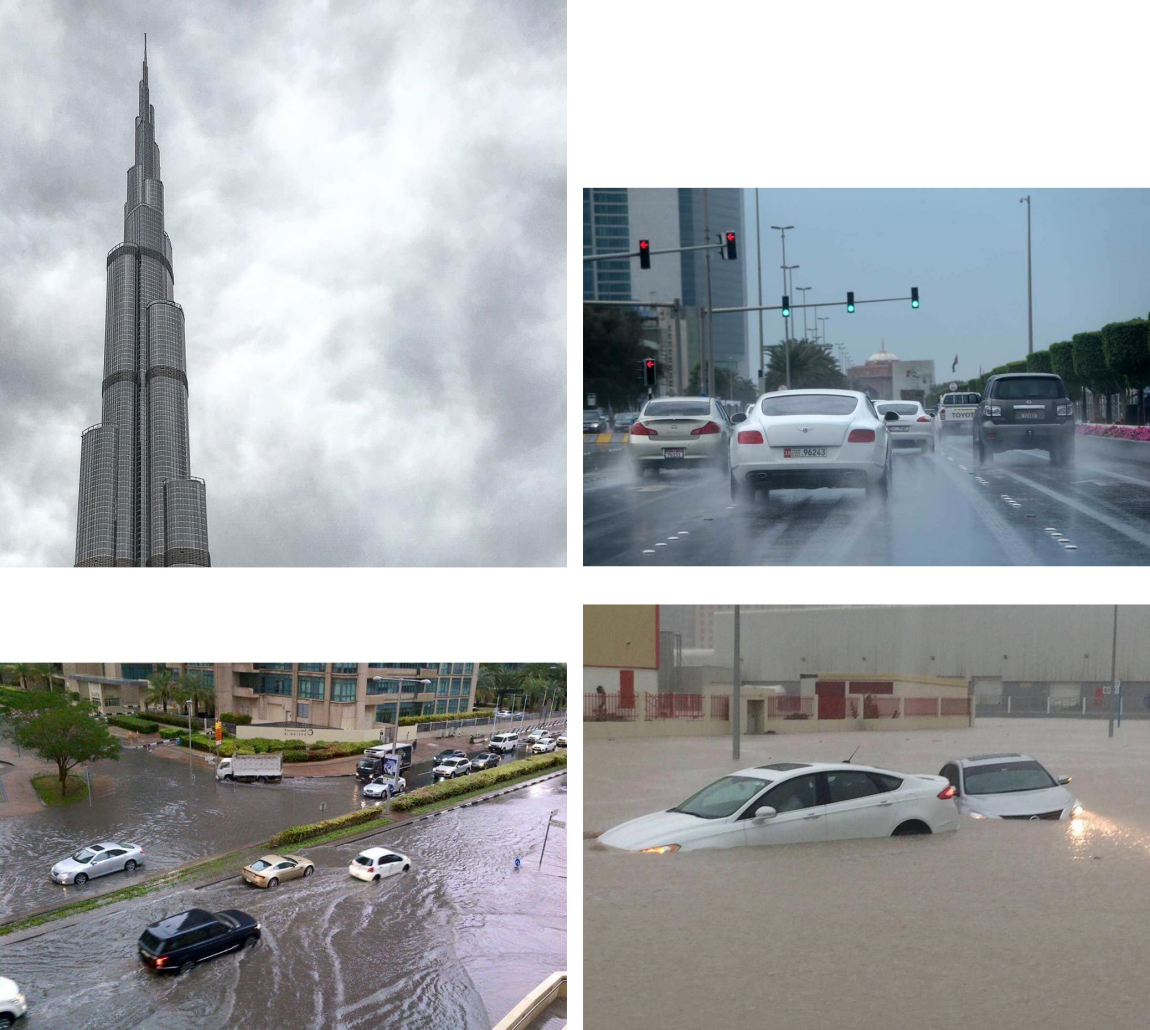
Sample images from final dataset containing classified images based on four classes (a) not relevant, (b) rain, (c) low flood and (d) high flood.

Fig 2 shows the sample images from the complete dataset, which were manually classified based on four classes (Table 2). The “irrelevant” class was assigned to images/videos that were irrelevant to our study but used the hashtags given in Table 1. For example, users who uploaded images of landmark buildings, clouds, swimming pools, and advertisements (commercial companies use weather-trending hashtags to sell food products during good weather) were assigned an irrelevant class.

The ResNet50 model is pre-trained on the ImageNet dataset and is available with the Keras API. ResNet50 was used as a transfer learning technique for CNNs to extract features. A Python code (S1 Appendix) was developed to (1) convert videos into frames and (2) extract features from converted frames and images.

**Fig 3:**
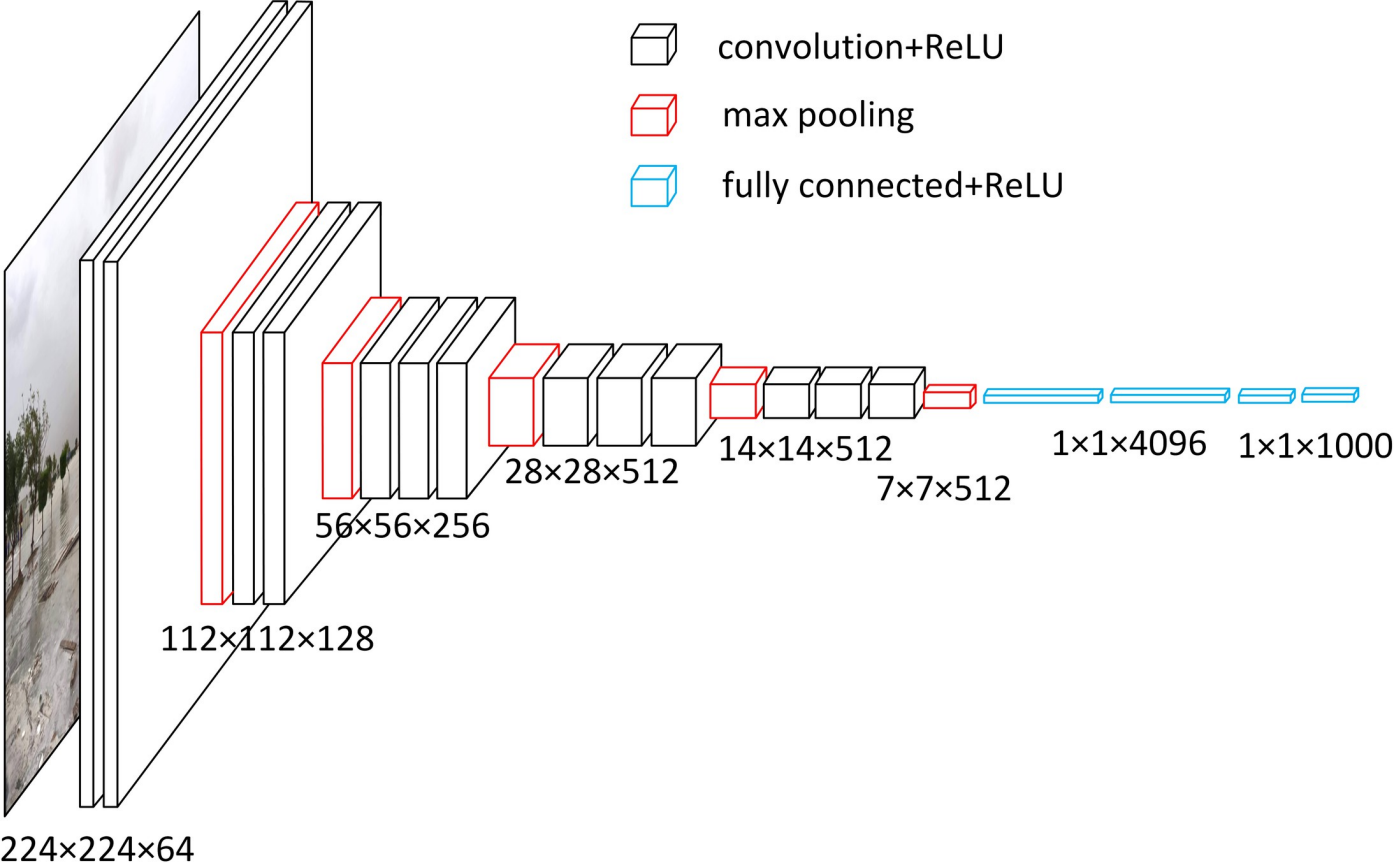
VGG-16 architecture for converting images into flattened features.

Feature extraction is based on VGG and ResNet architectures [44]. The VGG-16 feature extractor was initialized with the pertained weights created using ImageNet [45]. The architecture of VGG-16 has been largely used in visual data classification consisting of stacked convolution and max pooling layers (Fig 3). The input to the first layer is an RGB image with a size of 224 × 224. The image is then passed through different layers, which have filters with a very small receptive field of 3 × 3. The final layer is the SoftMax layer, which contains 1000 channels.

This process of feature collection for the identification of rain and floods in the images was executed using the programming of Keras with TensorFlow at the backend in the Anaconda Python environment. This was carried out to obtain features that could distinguish between different classes of our study. Each image and frame in the case of a video were converted into 1000 flattened features.

**Fig 4:**
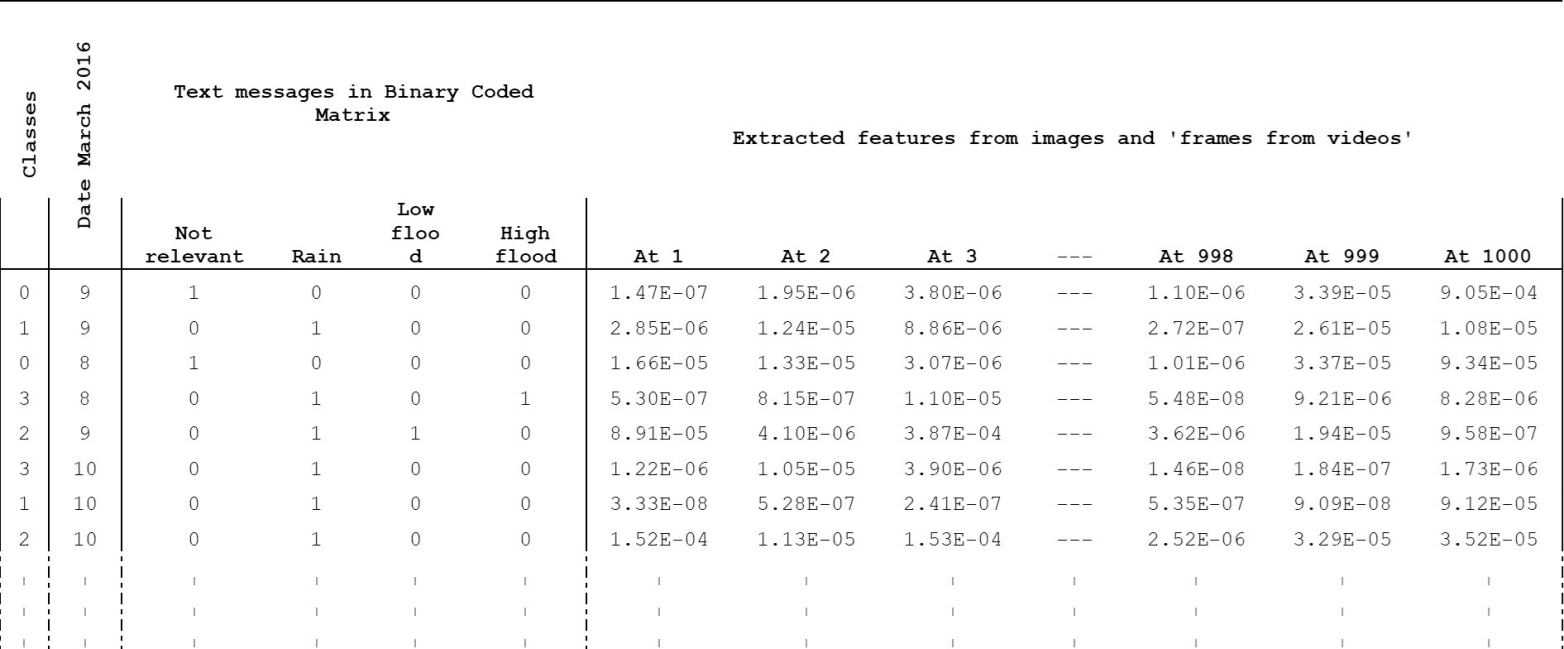
Sample data from 2,171 rows showing binary coded matrix of text messages and extracted features from images and frames.

The text messages associated with the videos and frames were also converted into a binary coded matrix for analysis in Weka. As the text message was uploaded on social media with image/video, they were organized in the same row along with their class (first column), which was assigned manually during image and video categorization (Fig 4).

**Fig 5:**
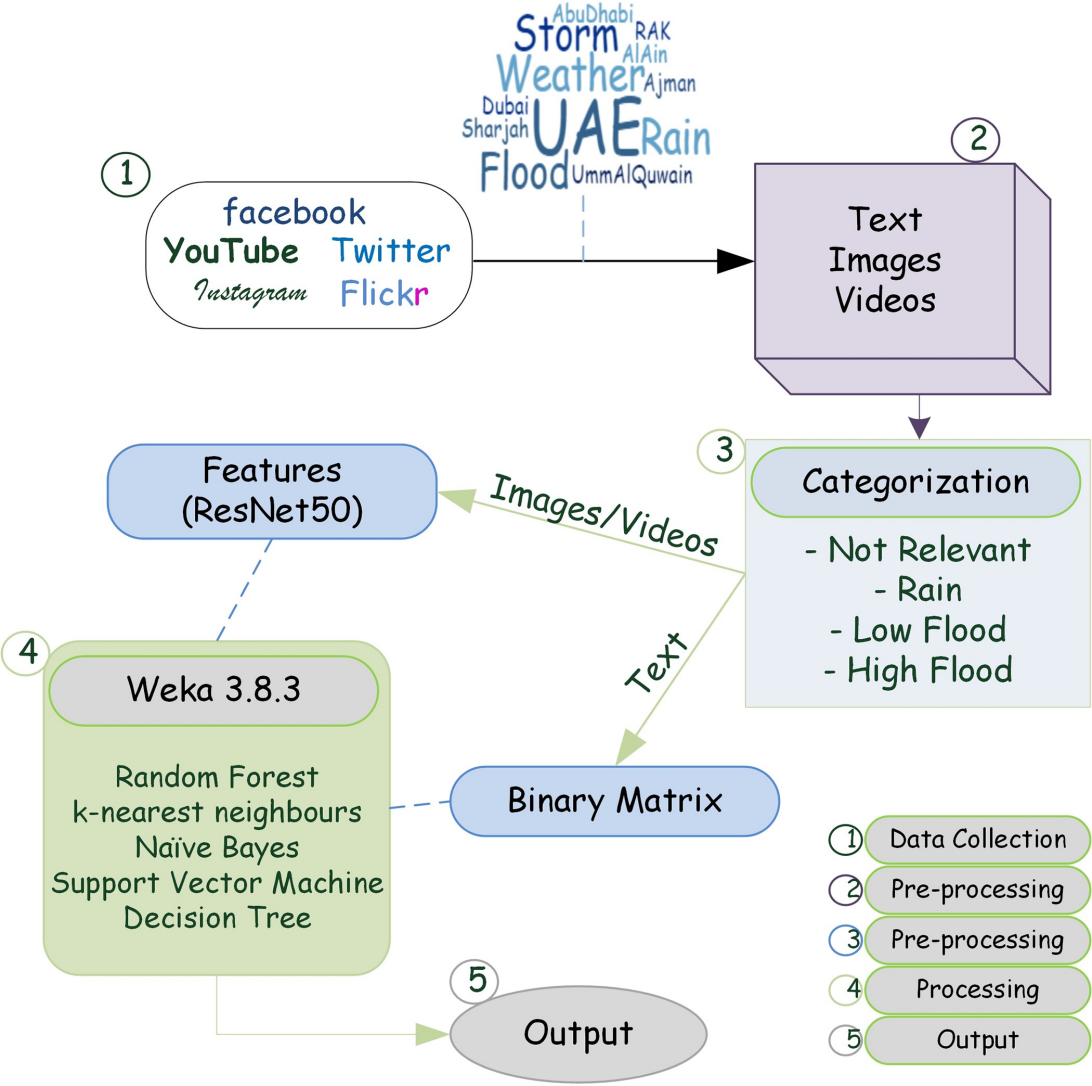
Methodology of the case study from data collection to output.

The features obtained from images and frames of video along with the text messages were classified based on four classes using the Weka tool (Fig 5). The data mining tool Weka, which was developed in the Java language was used in this study. This tool has many data-mining algorithms that are grouped into different groupings according to the rules generated by the algorithm. For this study, we used classification algorithms, namely random forest, k-nearest neighbors (IBk), naïve Bayes, support vector machine (SMO), and C4. 5 (J48). The test mode used was 20-fold cross-validation, and “full training set” was used as classifier model. The classification method involved 20-fold repetitions of the validation process 20 times.

The initial validation of study was conducted by comparing the activity of social media users with the precipitation/rainfall data from Global Precipitation Measurement (GPM). GPM, a climatic satellite, has many products, which are categorized into four levels by NASA. Level 0, 1, 2 and 3. Level-0 is raw unprocessed data which is used by Level-1 and produces brightness temperature. Level-2 uses Level-1 data and produces rainfall estimates. Whereas, Level-3 is recommended for use, as it is provided by Integrated Multi-satellite Retrievals (IMERG) algorithms and it combines all microwave, infrared satellite estimates and ground precipitation gauges [43]. Hence, this study used IMERG products (Level-3) data of precipitation. Since the data provided by IMERG is half-hourly precipitation (mm/30 min), the IMERG products were downloaded by accumulating half-hourly data to daily estimates for the period of March 7 to 11, 2016.

## Results and discussion

A total of 2171 rows containing 758 images and 1413 frames extracted from 154 videos, each having 1000 columns of attributes were analyzed and categorized into classes. Additionally, 705 texts from different online posts associated with these videos and images were also analyzed after converting them into a binary matrix. Different classifiers were used to test the data based on four different classes (irrelevant, rain, low flood, and high flood).

The model was set to run for three instances, first on the attributes of images and videos, then on the attributes of images and videos along with text, and finally, only on text. Table 3 shows the model accuracy and the time taken to build the model.

**Table 3:** Model accuracy from different functions in Weka.

Classifiers	Images & videos		Text, images & videos		Text	
	Accuracy	Time (sec)	Accuracy	Time (sec)	Accuracy	Time (sec)
Random Forest	80.18%	3.78	64.30%	1.23	61.28%	0.19
k-nearest Neighbours	76.22%	4E-3	52.94%	2E-2	60.99%	1E-3
Naïve Bayes	37.83%	0.44	41.38%	0.14	60.28%	1E-2
Support Vector Machine	69.82%	5.66	59.03%	0.98	62.84%	0.08
C4. 5 (J48)	72.07%	11.69	53.55%	1.94	63.12%	0.02

Result showed an interesting pattern where random forest gained the highest accuracy (80.18%), and Naïve Bayes had the lowest accuracy (37.83%) for the attributes of images and videos (Table 1). The text showed an accuracy of 61.28% using random forest, and the highest accuracy of 63.12% using C4. 5 (J48) classifier. When the text messages were combined with the attributes of video images, the accuracy of random forest dropped to 64.3%. This shows that the comparatively lower classified instances of text have reduced the accuracy from 80.18% to 64.3%.

For the execution time, k-nearest neighbors required 4E-3, 2E-2 and 1E-3 seconds for ‘images & videos’, ‘text, images & videos’ and ‘text’ respectively. The highest time taken to build the model was the C4. 5 (J48) as 11.69 s for images and videos whereas for the random forest, which showed highest accuracy for images and videos, took 3.78 s to build the model.

On the basis of accuracy and time while studying three instances for flood prediction, it was confirmed that random forest is the best classifier that gained the highest accuracy and comparatively less time to build the model on image and video attributes. This also showed that the information provided by users on social media in terms of text messages is less relevant. Additionally, the Naïve Bayes algorithm showed the least accuracy among all classifiers for all three instances, and hence, it cannot be used for the purpose of such classifications.

The scarcity of flow data is the result of a combination of conditions typically observed in arid areas, especially in the UAE. Almost all water courses on the Arabian Peninsula are ephemeral or intermittent in nature with water flow only for short periods of time. The region is generally sparsely populated (except in coastal areas), which makes regular maintenance visits as well as event-based visits challenging. Flood events typically occur in the form of flash floods because of high-intensity, short-duration rainfall events. Furthermore, the flood volume is often very high, which may cause the destruction of the flow gauge blocking of the gauge with large debris carried with the flood, or simply causing conditions that are too dangerous for manual flow gauges. Climatic conditions with extremely high temperatures during the summer may cause failure of the monitoring equipment.

**Fig 6:**
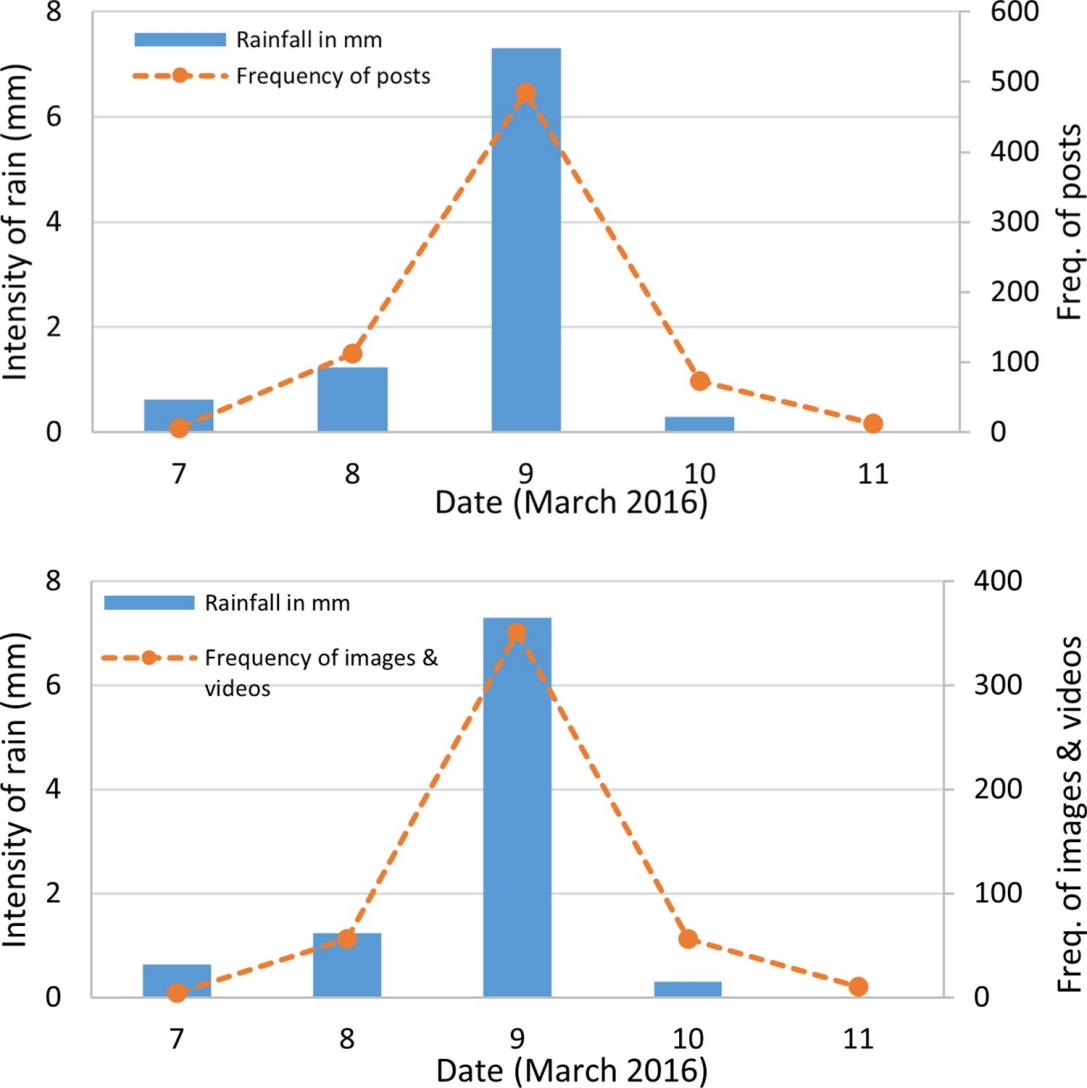
Time series of rainfall depths (a) with frequency of total posts per day, (b) with frequency of images and videos per day.

The rainfall data obtained from GPM for the UAE showed that rainfall started from March 7, 2016 was 0.63 mm and was the highest on March 9, 2016 at 7.3 mm. For the validation of data obtained from social media, two time series were plotted against the date. The number of online posts collected for this study from social media platforms were plotted on time series with the total number of posts (including text, images, and videos). Only ‘images and videos’ uploaded on selected dates are plotted against intensity of rainfall in mm. Evidence obtained from plotting these time series showed a significant relationship between rainfall and the frequency of posts, as well as the uploaded images and videos (Fig 6).

The actual rainfall event that triggered the flash flood was on March 9 and the frequency of posts was highest at 485. The same pattern was also observed for the images and videos uploaded on social media platforms, which were 351. This showed that social media users were more active on the day of the highest precipitation event. The activity on the hashtags of rain, flood, storm, and weather was highest on social media for this particular geographic location and the event.

Random Forest achieved the highest accuracy of 80.18% for the attributes of images and videos. Nair et al. [36] studied the frequency of social media users on the day of a flood in India and achieved the highest accuracy of 99.9% using random forest. However, the quality of social media data is never used before for studying floods. This study showed with reasonable classification accuracy, especially through Random Forest classifier, that machine learning algorithms can be effectively used to access visual and textual social media data for flood study.

### Models validation

The random forest performed best among all five classifiers, and the evaluation of all models is presented in Table 4. The kappa statistics showed the reliability of the model and that classification does not occur by chance [46]. The value of kappa statistics is 0.63, which is highest for images and video classification of random forest followed by KNN.

**Table 4:** Different classifier results for model accuracy, Kappa statistics, RMSE, F-measure, Area under Curve (AUC) and Precision Recall Curve (PRC).

Metrics		Accuracy	Kappa statistics	RMSE	F-Measure	AUC	PRC
**Random Forest**	Images & videos	80.18%	0.63	0.27	0.79	0.94	0.88
	Images, videos & text	64.30%	0.39	0.35	0.61	0.82	0.7
	Text	61.28%	0.08	0.37	0.54	0.65	0.58
**k-nearest neighbors**	Images & videos	76.22%	0.59	0.34	0.76	0.79	0.68
	Images, videos & text	52.94%	0.27	0.48	0.53	0.63	0.46
	Text	60.99%	0.08	0.39	0.54	0.63	0.56
**Naïve Bayes**	Images & videos	37.83%	0.18	0.56	0.4	0.68	0.52
	Images, videos & text	41.38%	0.22	0.54	0.43	0.66	0.47
	Text	60.28%	0.04	0.37	0.52	0.65	0.57
**Support Vector Machine**	Images & videos	69.82%	0.42	0.36	0.67	0.74	0.59
	Images, videos & text	59.03%	0.35	0.39	0.58	0.7	0.5
	Text	62.84%	0.01	0.39	0.77	0.5	0.45
**C4. 5 (J48)**	Images & videos	72.07%	0.53	0.36	0.72	0.78	0.65
	Images, videos & text	53.55%	0.23	0.47	0.53	0.64	0.45
	Text	63.12%	0.006	0.37	0.77	0.49	0.46

**Fig 7:**
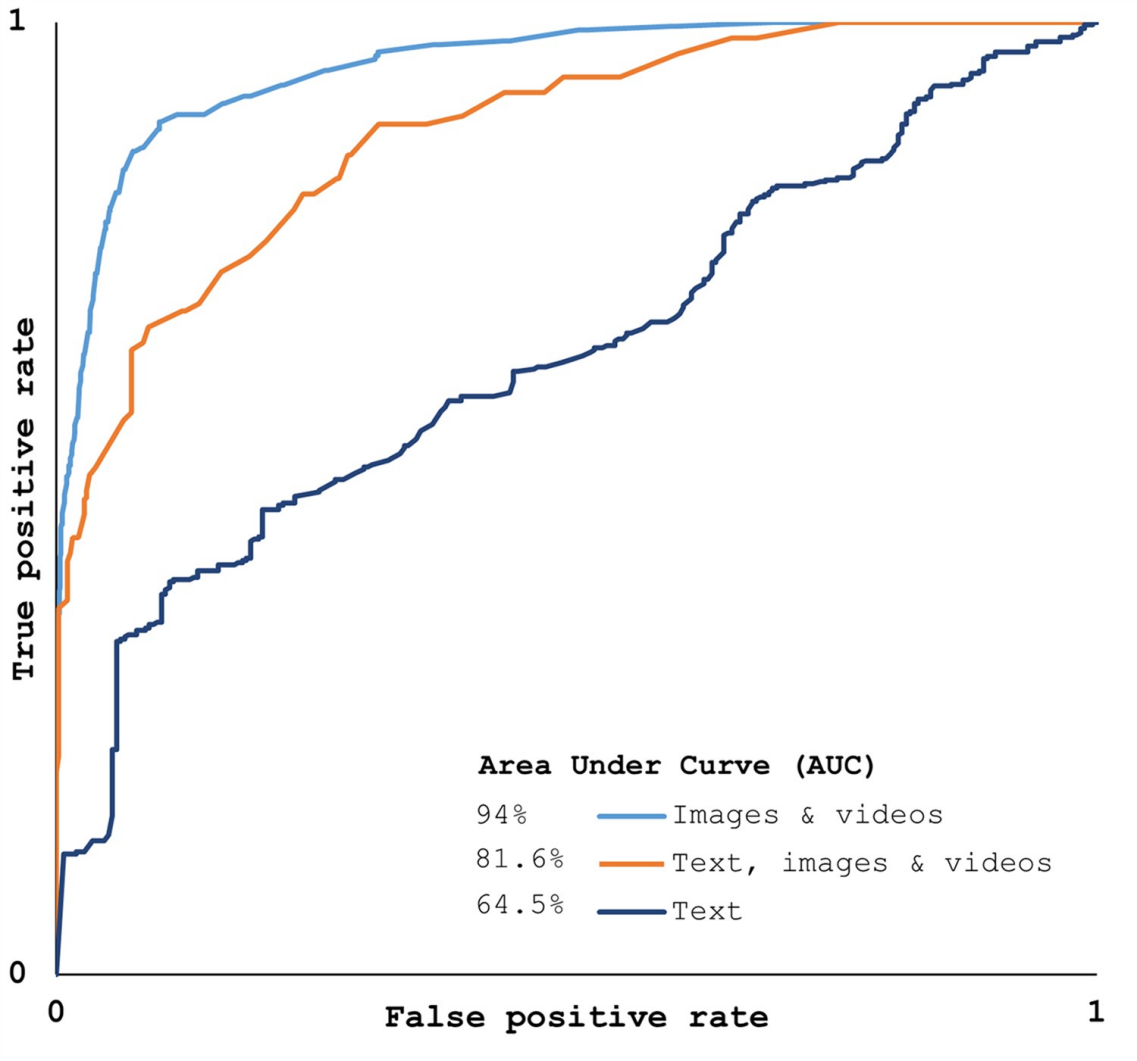
Area under Curve (AUC) for three set of data formats using random forest.

The root mean square error (RMSE) shows the difference between the observed values and model-predicted values [47]. The highest RMSE value (0.56) was recorded by Naïve Bayes and the least by Random Forest in image and video classification. Model performance can be evaluated using the AUC, with values lower than 0.5 indicating the inefficiency of the model, and values greater than 0.8 are considered good models [48]. The data formats of ‘images & videos’ and ‘images, videos, and text’ for the random forest classification showed the highest 0.8 AUC values, also plotted in Fig 7.

Since model evaluation only through AUC can be deceiving, precision recall (PRC) values are used to evaluate the sensitivity of the model along with positive predictive values [47]. The PRC value for ‘images and videos’ of random forest was the highest. These results showed that the accuracy and validity of random forest for images and videos were the highest among all other classifiers and data formats.

### Data quality assessment

The quality of the data obtained from social media is questionable. For instance, social media users usually add more hashtags, which could contain rain and floods in the same text, to get more viewership. This is reflected in the accuracy of this study, where text messages have comparatively low accuracy compared to images and videos. Additionally, other data quality issues are related to ‘irrelevant’ images and videos shared. For example, sharing an image of clouds or swimming pools with the hashtags of rain and floods reveals discrepancies. Also, social media users are not so technical to distinguish between low flood or high flood, which is replicated in the accuracy being comparatively lower than ‘images & videos’. To understand the quality of data on different social media platforms, data from Twitter, Facebook, Instagram, YouTube, and Flickr were evaluated separately using the random forest classifier (Table 5).

**Table 5:** Random forest classifier accuracy for data quality of different social media platforms.

Metrics		Random Forest					
		Accuracy	Kappa statistics	RMSE	F-Measure	AUC	PRC
**Twitter**	Images & videos	72.92%	0.52	0.32	0.71	0.87	0.79
	Images, videos & text	57.50%	0.3	0.37	0.6	0.75	0.62
	Text	41.25%	0.04	0.42	0.74	0.52	0.38
**Facebook**	Images & videos	80.46%	0.65	0.27	0.79	0.94	0.88
	Images, videos & text	66.67%	0.39	0.34	0.62	0.82	0.72
	Text	48.60%	0.01	0.42	0.37	0.46	0.35
**Instagram**	Images & videos	47.47%	0.11	0.38	0.36	0.69	0.54
	Images, videos & text	49.50%	0.12	0.38	0.53	0.72	0.57
	Text	45.50%	0.02	0.42	0.37	0.45	0.35
**YouTube**	Videos	83.61%	0.65	0.25	0.82	0.96	0.93
	Videos & text	40.91%	0.13	0.42	0.37	0.59	0.42
	Text	43.18%	0.21	0.43	0.42	0.59	0.41
**Flickr**	Images	74%	0.13	0.29	0.7	0.68	0.76
	Images & text	71.43%	0.16	0.33	0.5	0.69	0.73
	Text	57.15%	0.17	0.42	0.52	0.21	0.48

Video data from YouTube classified best with 83.61% accuracy followed by 80.46% for ‘image & videos’ data from Facebook. The AUC and PRC values for YouTube videos were also highest at 0.96 and 0.93, respectively. However, the classification of ‘Text’ from YouTube (video caption and description) achieved the lowest accuracy of 43.18% among all formats.

This is the first study in an arid region that analyzed data quality along with social media behavior usage during such flood events. The detailed social media data quality analysis—in terms of flood study, of each studied social media platform, under different formats of text, images and videos is explained in detail. Currently, there are no active flow monitors in the UAE. However, such flash floods are more common and are triggered by heavy precipitation. Hence, in such cases, the images and videos obtained from social media can be relied upon. Considering that flood data are very scarce across the UAE, such a database will be extremely valuable for public authorities concerned with flood management.

## Conclusions

An alternative method for flood analysis is suggested in this research, by proposing the use of social media data. This study aimed to investigate the quality of social media data for flood monitoring. Data related to flood events in the UAE of March 7 to 9, 2016 were collected from Facebook, Twitter, Instagram, YouTube, and Flickr. Results showed that Random Forest achieved highest accuracy with 80.18% for the data format of ‘images and videos.’ The binary codes of the text showed the least accuracy of 61.28%. The data from social media showed a significant correlation with rainfall data from the general circulation model and the number of posts mentioning flood-related keywords. The performance of the machine learning models was evaluated using the AUC and PRC. Random Forest also showed the highest AUC of 94% and PRC of 88% for ‘images and videos’. The data quality showed that Random Forest classified YouTube data of videos with highest accuracy, followed by ‘images & videos’ of Facebook, Flickr, Twitter and Instagram. These results indicated that the quality of images and videos from social media can be used for flood monitoring purposes by public authorities, especially in areas of the UAE with no active flow gauges.

The collection stage of the data was extremely intensive and time consuming due to the use of multiple hashtags and on different social media platforms. The application developed in S1 Appendix can be further enhanced by automating the input of data from social media platforms using different Application Programming Interface (APIs). Furthermore, the study of quality of social media data can be further advanced by using artificial intelligence and deep learning models to measure the flood water level in images and videos.

## Supporting information

S1 Appendix Python code for frames extraction from videos and conversion of images into features. (DOCX)
