# Supplementary material for: Utilization of social media in floods assessment using data mining techniques
Source: PLoS One. 2022 Apr 25;17(4):e0267079. doi: 10.1371/journal.pone.0267079 (PMC9037947; doi:10.1371/journal.pone.0267079)
Supplement: S1 Appendix — (DOCX) [file pone.0267079.s001.docx]

# Appendix A

Python code for frames extraction from videos and conversion of images into features

import cv2

import os

from keras.preprocessing import image

from keras.applications.vgg16 import VGG16

from keras.applications.vgg16 import preprocess_input

import numpy as np

'''This function extracts frames from videos and save them as .jpg file'''def extractVideoToFrames():

for entry in os.scandir('.'):

if entry.is_file():

if entry.name.lower().endswith(".mp4"):

vidcap = cv2.VideoCapture(entry.name)

success, image = vidcap.read()

count = 0

success = True

while success:

vidcap.set(cv2.CAP_PROP_POS_MSEC, (count * 15000))

cv2.imwrite(entry.name+"frame%d.jpg" % count, image) # save frame as JPEG file

success, image = vidcap.read()

print('Read a new frame: ' + str(success))

count += 1

'''This function extracts features from frames using VGG16 architecture'''

def extractFeaturesFromFrame():

for entry in os.scandir('.'):

if entry.is_file():

if entry.name.lower().endswith(".jpg"):

features = extract_resnet(entry.name)

np.savetxt(str(entry.name)+"features.csv", features, delimiter=",")

def extract_resnet(img_path):

_image_h = 224

_image_w = 224

model = VGG16(weights='imagenet')

img = image.load_img(img_path, target_size=(_image_h, _image_w))

x = image.img_to_array(img)

x = np.expand_dims(x, axis=0)

x = preprocess_input(x)

features_array = model.predict(x)

return features_array

if __name__=="__main__":

extractVideoToFrames()

extractFeaturesFromFrame()
